# Benefits and problems of electronic information exchange as perceived by health care professionals: an interview study

**DOI:** 10.1186/1472-6963-11-256

**Published:** 2011-10-07

**Authors:** Marieke Zwaanswijk, Robert A Verheij, Floris J Wiesman, Roland D Friele

**Affiliations:** 1 NIVEL, Netherlands Institute for Health Services Research, P.O. Box 1568, 3500 BN Utrecht, the Netherlands; 2 Department of Medical Informatics, University of Amsterdam/Academic Medical Centre, Amsterdam, the Netherlands; 3 Faculty of Social and Behavioural Sciences, Tilburg University, Tilburg, the Netherlands

## Abstract

**Background:**

Various countries are currently implementing a national electronic patient record (n-EPR). Despite the assumed positive effects of n-EPRs, their overall adoption remains low and meets resistance from health care providers. This study aims to increase our understanding of health care providers' attitude towards the n-EPR, by investigating their perceptions of the benefits and problems of electronic information exchange in health care and the n-EPR in particular.

**Methods:**

The study was conducted in three Dutch health care settings: acute care, diabetes care, and ambulatory mental health care. Two health care organisations were included per setting. Between January and June 2010, interviews were conducted with 17 stakeholders working in these organisations. Relevant themes were deduced by means of thematic qualitative analysis.

**Results:**

Health care providers perceived electronic information exchange to promote the efficiency and quality of care. The problems they perceived in electronic information exchange mainly concerned the confidentiality and safety of information exchange and the reliability and quality of patient data. Many problems perceived by health care providers did not specifically apply to the n-EPR, but to electronic information exchange in general.

**Conclusions:**

The implementation of the Dutch n-EPR has mainly followed a top-down approach, thereby neglecting the fact that the perceptions and preferences of its users (health care providers) need to be addressed in order to achieve successful implementation. The results of this study provide valuable suggestions about how to promote health care providers' willingness to adopt electronic information exchange, which can be useful for other countries currently implementing an n-EPR. Apart from providing information about the benefits and usefulness of electronic information exchange, efforts should be focused on minimising the problems as perceived by health care providers. The safety and confidentiality of electronic information exchange can be improved by developing tools to evaluate the legitimacy of access to electronic records, by increasing health care providers' awareness of the need to be careful when using patient data, and by measures to limit access to sensitive patient data. Improving health care providers' recording behaviour is important to improve the reliability and quality of electronically exchanged patient data.

## Background

For several years, the Dutch government has been preparing to implement a national electronic patient record (n-EPR) for the entire health care sector (see additional file 1: The Dutch n-EPR). The n-EPR is meant to improve health care providers' access to relevant patient data, under the assumption that this will lead to improvements in the efficiency, continuity, safety and quality of care [1,2]. Similar developments are taking place in other countries worldwide (e.g. the UK, Canada, the US, Australia and France) [1,3].

Despite the assumed positive effects of n-EPRs, their overall adoption remains relatively low and meets resistance from health care providers [1]. One third of Dutch health care professionals, for instance, have shown reluctance to accept the n-EPR as proposed by the Dutch government [4].

Health care providers' perceptions and preferences about the n-EPR are likely to affect their willingness to adopt it. The importance of users' perceptions in the introduction of new technologies is underscored in the well-known Technology Acceptance Model and its adaptations [5-7]. Studies investigating this model in health care have consistently shown perceived usefulness (i.e. the degree to which a person believes that using a particular technology will enhance his or her job performance) to be a strong determinant of usage intentions [8-10]. However, the model has been criticised for its one-sided focus on factors that facilitate the adoption of technology, while neglecting factors that may hinder adoption, such as users' negative perceptions or their resistance to change [8]. Gaining insight into positive as well as negative perceptions of health care providers is crucial to fully understand their attitude towards the n-EPR.

As many Dutch health care providers already exchange patient data by means of local or regional electronic information systems, their reluctance to adopt the n-EPR is quite remarkable. The question arises, therefore, whether this reluctance is associated with electronic information exchange in general, or with specific attributes of the n-EPR.

This study aims to increase our understanding of health care providers' attitudes towards the n-EPR, by investigating their perceptions of the benefits and problems of electronic information exchange in health care. We will distinguish between perceived problems that are specifically associated with the n-EPR and problems that apply to electronic information exchange in general. The results will provide valuable suggestions about how to promote health care providers' trust in and willingness to adopt electronic information exchange, which can contribute to an effective implementation of the n-EPR.

## Methods

The study was conducted in three health care settings which are particularly demanding with respect to the exchange of patient information. First, the acute care setting was selected because of its requirements for the completeness of information and the speed of information exchange. Secondly, diabetes care was included, because the multidisciplinary character of this kind of care causes information exchange between the various involved health care providers to be crucial. Thirdly, ambulatory mental health care was selected, because privacy issues are likely to play an important role in this setting. This may cause patients to object to the inclusion of information in electronic files that can be accessed by other health care professionals. It may also cause health care providers to be reluctant to record certain information about patients in electronic records.

To obtain a broad picture of health care providers' perceptions of electronic information exchange, we aimed to include two health care organisations in each setting that differed in the extent to which electronic information exchange was being used. Several stakeholders within the health care sector were contacted to obtain information about the degree of implementation of electronic information exchange in health care organisations. This information was used to select health care organisations, which were asked to participate in the study. In each of the three settings, two health care organisations were included, leading to six case studies.

The aim of including in each setting two health care organisations that differed in the extent to which electronic information exchange was being used was not met for ambulatory mental health care. Although several mental health care organisations with fairly low degrees of implementation of electronic information exchange were approached, none of them agreed to participate. Therefore, two mental health care organisations with relatively high degrees of the use of electronic information exchange were included.

Data were collected by means of interviews. This method allows respondents to express their individual perceptions and thereby produces an in-depth understanding of the topic. Contact details of relevant stakeholders whom we could ask to participate in an interview were requested. In total, 21 stakeholders were asked to participate. They received written information about the study. Four of them declined because of a lack of time. In each health care organisation, two or three stakeholders consented to be interviewed, resulting in a total number of 17 interviews.

Respondents' opinions about the benefits and problems of electronic information exchange and the n-EPR were investigated using a predetermined topic list (see additional file 2: Topic list used in the interviews). For each health care setting, a scenario describing a patient's contact with the health care organisation was constructed. Based on these scenarios, we investigated how information was being exchanged in the organisation and which problems were encountered.

Data collection took place between January and June 2010. Two authors (MZ and FW) performed the interviews. To ensure comparability in the way the interviews were performed, five interviews (distributed over the total period of data collection) were performed by the two authors together. The remaining interviews were performed by one of the interviewers separately (8 by MZ; 4 by FW). Non-directive interview techniques were used to minimise the risk of biasing respondents' answers. Sixteen interviews were conducted in the health care organisation, one interview was conducted in the respondent's home. Interviews lasted 85 minutes on average. An audit trail was maintained throughout the study. Characteristics of the respondents are presented in Table 1.

**Table 1:** Characteristics of respondents (N = 17)

	Respondents		Degree of exposure to electronic information exchange^1^	
	
	N	%	High (N = 12)	Low (N = 5)
**Health care setting**				
Acute care	6	35	3	3
Diabetes care	5	29	3	2
Ambulatory mental health care	6	35	6	-
**Function**				
GP	7	41	3	4
GP assistant	2	12	1	1
Psychologist/social worker	2	12	2	-
Psychiatrist	2	12	2	-
Medical informatics expert	2	12	2	-
Other	2	12	2	-
**Gender**				
Male	12	71	8	4
Female	5	29	4	1

^1 ^Based on the extent to which electronic information exchange is being used in the participating health care organisation

Interviews were audio taped and transcribed, and identifying details of respondents were removed from the transcripts. Relevant themes were deduced by the first author by means of thematic qualitative analysis [11]. The resulting coding scheme was discussed within the research team. Disagreements were discussed until consensus was achieved. Several data verification procedures were used, including concurrent data collection and analysis, and idea reconfirmation during the process. To validate the results, they were discussed within the research team and within an advisory group of national and international experts in the fields of health care and health care legislation.

The inclusion of respondents was discontinued when theme saturation was observed. Theme saturation was determined by analysing the data of thirteen interviews. Subsequently, data from four additional interviews were analysed, which revealed no new themes.

The researchers were not in any way involved in the implementation of the n-EPR. It is therefore unlikely that the results have been biased in this respect.

## Results

The respondents stressed the general importance of having access to patient information from other health care providers, whether exchanged by telephone, on paper or electronically. Although the majority of respondents indicated that they did not rely blindly on information provided by others, they regarded the information as particularly useful as a starting point for further examination or to reveal patterns in patients' behaviour or health.

In the following paragraphs, respondents' perceptions of the benefits and problems of electronic information exchange will be described. Interview results are presented with illustrative quotations that reflect supportive and deviant cases. A summary of the results is presented in Table 2.

**Table 2:** Perceived benefits and problems of electronic information exchange

Perceived benefits	
Improvements in the efficiency of health care and the speed of communication	
Access to up-to-date information about patients	
Improvements in the quality of care (e.g. prevention of medical errors)	

**Perceived problems: confidentiality and security of electronic information exchange**	
**General**	**n-EPR**

- Unauthorised persons having access to patient data due to limited security of electronic information systems or carelessness/misuse by health care providers	- Risks regarding patients' privacy and unauthorised persons' access to patient data will increase
- Using authorisation profiles to organise access to electronic patient records can cause problems in crisis situations	- Limited safety of the UZI-pass
- The limited usefulness of logging data for evaluating the legitimacy of health care providers' access to patient data	- It is unclear how the legitimacy of health care providers' access to patient data will be evaluated and who will be responsible for the evaluation
	- Essential information may be missed because of patients' desire to protect their privacy and conceal their electronic records or parts of it

**Perceived problems: reliability and quality of patient information**	

- Inadequate recording of patient information	- Inadequate recording of patient information
- Interoperability problems between information systems	- Health care providers may become more cautious in recording sensitive or personal patient information in electronic records
- Information overload	- Doubts about the technical performance of the n-EPR
- Limited speed of electronic information exchange	

**Other perceived problems**	

- Health care providers' liability in case of medical errors is unclear	- Unfamiliarity of other health care providers
- Limited usefulness of protocols and guidelines	

### Perceived benefits of electronic information exchange

According to respondents in all settings, electronic information exchange can improve the efficiency of care and the speed of communication. For instance, time can be saved when information can immediately be imported into the recipient's electronic information system and when referrals or requests for laboratory tests can be exchanged electronically rather than on paper.

The efficiency of care was also perceived to improve because electronic information exchange allows health care providers to have access to up-to-date information about patients, which is useful to prevent duplicate testing or when patients are unable to indicate their problems themselves, for instance because they are unconscious or confused. This benefit was mainly reported by respondents working in diabetes care and acute care.

Some respondents indicated that electronic information exchange can improve the quality of care. By enabling access to up-to-date information about patients, medical errors may be prevented. For instance, exchanging information about medication use may help to detect counter-indications or allergies. This benefit was reported in all three included health care settings.

### Perceived problems of electronic information exchange in general

The respondents reported various problems of electronic information exchange in general, which can apply to information exchange on a local, regional or national level. These problems will be described first, followed by the perceived problems that were specifically associated with the n-EPR.

In describing the perceived problems, we distinguish between problems associated with the confidentiality and security of electronic information exchange, problems associated with the reliability and quality of patient information, and other problems.

#### Problems associated with the confidentiality and security of electronic information exchange

Respondents from all three health care settings expressed concerns about the safety of information systems and information exchange. They were concerned that unauthorised persons would have access to electronic patient data, either because people could hack the system, or as a result of health care providers' carelessness (e.g. leaving their computer screen unattended or using insecure e-mail connections to exchange patient information). They also indicated that health care providers could misuse their access to patient records to browse through the information of other patients. However, they also realised that: *'A 100% secure system is not workable. It'll be so user-unfriendly that it'll be impossible to work with. So it's always a compromise between user-friendliness and safety' *(GP, acute care).

In most participating health care organisations, access to patient records was organised by authorisation profiles, which were based on health care providers' job position and location. The use of authorisation profiles may cause problems in crisis situations, when health care providers may need access to patient data they normally would not be allowed to have access to.

Evaluating the legitimacy of health care providers' access to patient data poses problems. Because all accesses to patient records are logged, the amount of logging data produced each day makes it almost impossible to evaluate the data on a standard basis. The logging data are usually evaluated only when unauthorised access is suspected. Respondents therefore doubted the usefulness of logging data to ensure patients' privacy.

#### Problems associated with the reliability and quality of patient information

##### 1. Recording of patient information

The respondents emphasised that electronic information exchange increases the quality requirements imposed on patient records. Whereas these records previously served merely as a mnemonic device for health care providers, nowadays an increasing number of other health care providers have to be able to use and understand the recorded information. Good quality recording is therefore crucial. However, respondents from all settings reported that patient data are not always recorded adequately, which may cause essential information to be missed or misunderstood.

Despite an increasing attention for adequate record keeping and adequate use of diagnosis codes (e.g. by the development of guidelines) [12], respondents working in diabetes care and acute care indicated that some health care providers did not use codes to record diagnoses or complaints, but instead recorded them as free text: *'If an episode is titled 'finger' or 'knee', you still don't know what's wrong' *(GP, acute care). Others used these codes inadequately (e.g. not recording recurrent episodes of the same diagnosis under the same code), which may prevent other health care providers from getting a coherent picture of the patient's problems.

Respondents also indicated that the structure of some information systems causes patient data to be recorded in wrong parts of the information system, which makes it difficult for others to find relevant information.

##### 2. Interoperability problems

The limited interoperability (i.e. the ability to exchange and use information) between information systems was perceived as a major threat to adequate electronic information exchange in the three included health care settings.

Firstly, some information systems are not interoperable at all. In these cases, information is exchanged by regular mail or fax. The printed information subsequently needs to be imported in the recipient's information system, by adding a scanned version to the information system or by typing a summary in the system. This entails problems of inefficiency and the risk of making errors.

Other information systems are in principle interoperable. However, the recipient's information system not always adequately records the received patient data. Information may be placed in the wrong part of the system or may be missing all together. Health care providers therefore do not know whether relevant information is missing from their information system and if so, which information is missing. *'Every time I use information, I have to keep in mind: ok, I see some information, but I might not see everything' *(GP, acute care).

##### 3. Information overload

Respondents from ambulatory mental health care indicated that they suffered from information overload, which makes it difficult to find the information that is essential for the provision of high quality care. Structural limitations of the information systems used in some organisations cause the majority of patient data to be recorded in the same part of the information system, without any distinction between relevant and less relevant information. A related issue is the fact that ambulatory mental health care, unlike general practices and out-of-hours services, does not use summary care records containing only the most essential information, when exchanging information with other health care providers.

Respondents from ambulatory mental health care not only perceived an overload in the information to *digest*, but also experienced a burden of having to *produce *too much information. They perceived the time needed to record patient data in electronic records as a problem, because it limited the time available for patient contacts. It also limited the amount of detail recorded in electronic patient records, which may negatively affect the quality of the information.

##### 4. Technical performance of information systems

Most respondents indicated that technical breakdowns, causing information systems to be temporarily unavailable, seldom occurred. They did, however, experience some problems regarding the speed with which information could be exchanged. Particularly logging into the electronic information system sometimes took too much time, causing patient information to be unavailable during consultations. This problem was mainly reported in acute care.

#### Other perceived problems of electronic information exchange in general

##### 1. Health care providers' liability

The respondents found it unclear who is liable when medical errors are made because of incompleteness or incorrectness of electronically exchanged patient information.

In case of regional or national electronic information exchange, it is not always clear which health care provider has added information to a patient record. Moreover, health care providers may not be aware that relevant information is missing in patient records, because of inadequate interoperability between information systems or patients' desire not to disclose certain information. These issues make it difficult to trace the cause of errors and therefore complicate liability issues. This problem was mainly mentioned by respondents from acute care.

##### 2. Limited usefulness of protocols and guidelines

Although the participating organisations used protocols and guidelines to guide the electronic exchange of patient information, respondents - mainly from ambulatory mental health care - were not familiar with their specific content. They ascribed their lack of knowledge to the limited comprehensibility and usefulness of protocols and guidelines, which, in their opinion, provided too few suggestions for their application in daily practice.

Merely one respondent (acute care) expressed a different opinion: she stressed the usefulness of protocols and guidelines for following standard procedures and preventing errors. She felt that the guidelines were applicable in daily practice, mainly because they were regularly adapted to suggestions of their users.

### Perceived problems specifically associated with the n-EPR

Apart from the perceived problems and threats mentioned above, which can apply to electronic information exchange on a local, regional or national level, the respondents described several problems which were specifically associated with the n-EPR.

#### Problems associated with the confidentiality and security of electronic information exchange

Most respondents from the three included health care settings perceived safety problems to increase in case of an n-EPR: *'If someone succeeds in breaking into the National Switch Point, I'm afraid that it'll be over. That's my biggest fear, that there'll be leaks or that people will be careless' *(GP, acute care). Respondents were concerned about patients' privacy because of too many health care providers having access to patient data. Although this risk may also exist in regional electronic information exchange, it was mainly mentioned in relation to the n-EPR.

In the n-EPR, access to patient records is restricted to health care providers who are directly involved in the care for a particular patient. Respondents indicated that it is unclear how this concept will be operationalised and who will be responsible for evaluating whether health care providers have rightfully accessed patient records.

As described in additional file 1, health care providers can get access to the n-EPR by means of a personal ID chip card (the so-called UZI pass, which is a Dutch abbreviation for 'unique health care provider identification pass') and password. Respondents from acute care were worried about the safety of the UZI pass and indicated that it had already been hacked. The safety of patient data may also be compromised because health care providers did not always use their pass with adequate caution. As a respondent put it: *'The weakness of the UZI pass is its user' *(GP, acute care).

The larger number of persons having access to patient records in case of an n-EPR may cause health care providers to become more cautious in recording sensitive patient information in electronic records: *'My recording of patient information will definitely change if others will have access to my patient records. Information about personality disorders and violence or rape, with names and all, will just be left out' *(GP, diabetes care).

Although patients' privacy may be threatened by electronic information exchange, the quality of electronically exchanged information may, on the other hand, be threatened by patients' desire to protect their privacy. In the n-EPR, patients will have the right to conceal their records or parts of it. This may cause health care providers to miss essential information. Since they are not allowed to ask if patients have concealed any part of their record, they do not know *if *any information is missing, let alone *which *information is.

#### Problems associated with the reliability and quality of patient information

##### 1. Recording of patient information

Inadequate recording of patient data was also perceived as a major problem of the n-EPR: '*I worry about the quality of recording at a national level. I've been worrying about that for years. The quality has been miserable for years now. It's an illusion to think that it'll improve quickly' *(GP, acute care).

Some respondents indicated that their recording behaviour had changed because of electronic information exchange. They expected this effect to be even stronger in case of an n-EPR. This could on the one hand have a negative effect on the quality of patient records, because health care providers may want to protect their patients' privacy and may therefore become more cautious in recording sensitive patient information. It may, on the other hand, improve the quality of patient records: *'Since I knew that health care providers of out-of-hours services had access to information about my patients, I've changed my way of recording. Nowadays, I ask myself: will someone else understand what I've written here?' *(GP, acute care)

In the light of these findings, several respondents emphasised that health care providers should not blindly trust the information provided by others: *'Some people state that patients don't have to tell their story over and over again in case of electronic information exchange. That's a dangerous opinion, because patients do have to tell their story each time they consult a health care provider. Medical errors occur by relying too much on information provided by others' *(GP, acute care).

##### 2. Technical performance of information systems

Some respondents expressed doubts about the technical performance of the n-EPR: *'Technically it hasn't functioned adequately anywhere yet. So it remains to be seen if it'll work and within which time frame it will' *(GP, acute care).

On the other hand, one respondent (diabetes care) argued that the implementation of the n-EPR would improve the technical quality of electronic information systems, because connection to the national infrastructure is only allowed when certain quality requirements have been fulfilled (see additional file 1).

##### 3. Not being personally acquainted with other health care providers

Respondents from the three included health care settings perceived the unfamiliarity of other health care providers as a problem of electronic information exchange. This was mainly perceived as a problem of the n-EPR, since most respondents felt that they were personally acquainted with health care providers within the region. One respondent expressed another view: *'It's nonsense to argue that you're familiar with people within the region. Even at a regional scale, I have to deal with many different people. I don't know them all' *(GP, acute care).

Respondents generally had more trust in the information they received from health care providers they personally knew. Based on previous experiences with these health care providers, they could better value the adequacy of the information. They also found it easier to contact familiar health care providers when they had questions concerning the received information.

## Discussion

Although n-EPRs are assumed to improve the efficiency, continuity, safety and quality of care, their implementation has been hampered by resistance of health care providers in several countries [1]. To increase our understanding of health care providers' attitudes towards the n-EPR, this study investigated their perceptions of the benefits and problems of electronic information exchange in general and the n-EPR in particular. Insight into these topics can provide suggestions about how to promote health care providers' willingness to adopt electronic information exchange, which can be useful for other countries currently implementing an n-EPR.

Many of the problems perceived by health care providers did not specifically apply to the n-EPR, but to electronic information exchange in general. Apparently however, these perceived problems do not negatively affect health care providers' willingness to use electronic information exchange on local or regional levels. The fact that many Dutch health care providers already exchange patient data by means of such local or regional electronic information systems, may indicate that they acknowledge the importance and usefulness of electronically exchanging patient information. Indeed, the health care providers in this study perceived several benefits of electronic information exchange, which can be regarded as aspects of perceived usefulness as defined in the Technology Acceptance Model [5,6]. They expected electronic information exchange to promote the efficiency and quality of care. These results support previous studies, in which perceived usefulness has been found to positively influence health care providers' intentions to use new technologies [8-10].

Despite the perceived benefits of electronic information exchange and health care providers' willingness to use electronic information exchange on local and regional levels, they show reluctance to adopt the n-EPR [4]. This apparent contradiction may be explained by differences in the development of local and regional systems compared to the n-EPR. The first have been developed in a decentralised bottom-up way, led by the initiatives and needs of local health care providers. In contrast, the introduction of the Dutch n-EPR has followed a centralised approach. This large-scale project was initiated by the Dutch government and has mainly been implemented in a top-down way. Such an approach is required to execute macro level changes, such as implementing a central infrastructure or developing national legal regulations [13]. However, the top-down approach as used in the introduction of the n-EPR is bound to fail without the commitment and acceptance of its primary users, the health care providers [13]. This was confirmed in several publications, in which health care providers explicitly expressed their discontent with the top-down approach of the Dutch government [e.g. [4,14]]. Health care providers' perceptions and preferences about electronic information exchange are likely to affect their willingness to adopt the n-EPR and therefore need to be addressed to achieve successful implementation.

Other factors may underlie the perceived problems found in this study. For instance, health care providers' reluctance to adopt the n-EPR may stem from their reluctance to change their established way of working or from fear of losing their professional autonomy [1,15]. Alternatively, their reluctance could be due to the timeframe difference in the implementation of local/regional systems and the n-EPR. Health care providers have had more time to get used to local and regional electronic information exchange than to the n-EPR.

Based on the results of this study, several measures to promote the implementation of the n-EPR can be suggested. First, providing additional information about the benefits and usefulness of electronic information exchange, for instance by using local opinion leaders, may be a fruitful way to stimulate successful implementation [9,16]. This approach is hampered, however, by a lack of evidence about the effects of electronic information exchange on the efficiency, quality and costs of health care [1,2,17,18]. More research on these topics is therefore needed.

The Technology Acceptance Model explains the adoption of new technologies by merely focusing on factors that facilitate adoption, such as users' perceptions of the usefulness of the new technology. The model has been criticised for neglecting factors that may hinder adoption, such as users' negative perceptions or their resistance to change [8]. Therefore, merely emphasising positive aspects of electronic information exchange will not be sufficient to promote implementation of the n-EPR. Indeed, information about the n-EPR provided by the Dutch government has been criticised by health care providers and patients to be positively biased [14].

Efforts should also be focused on minimising the problems that health care providers perceive in electronic information exchange, as these may cause resistance to implementation [19]. Based on our results on perceived problems, several measures to promote the implementation of the n-EPR can be suggested.

### Confidentiality and security of electronic information exchange

In line with previous findings [2], health care providers were concerned about the confidentiality and security of electronic information exchange. These concerns were mainly mentioned in the context of the n-EPR. The amount of data being available through the n-EPR and the number of included health care providers indeed increase the risk of unauthorised access to patient data. Although the n-EPR contains more elaborate security requirements than currently applied in local and regional information exchange [20], doubts have been expressed whether the national requirements sufficiently protect against unauthorised access to patient data [21]. At this moment, the legitimacy of access can only be evaluated by means of logging data, i.e. *after *possible unauthorised access has occurred. Because of the large amount of logging data produced each day, finding ways to automatically select suspicious requests for access is crucial to facilitate the monitoring of access to patient data.

Apart from such technical measures, increasing health care providers' awareness of the importance of carefulness in using and accessing patient data (e.g. carefully using their UZI pass) may improve the safety and confidentiality of electronic information exchange. Particularly respondents from acute care expressed doubts about the safety of the UZI pass. This may have to do with the stepwise approach followed in the implementation of the Dutch n-EPR (see additional file 1), which has caused health care providers in acute care to be most experienced in using the UZI pass.

To safeguard patients' privacy, some diagnoses (e.g. abuse or sexually transmitted diseases) can be given a privacy code, which limits the accessibility of these data to others. Another possibility is to record these data in a separate part of the information system, which is only accessible to a limited group of health care providers.

Giving patients the responsibility to manage their own data (e.g. by providing a chip card that contains essential personal health information, or by securing a patient record with the patient's own password) may also prevent privacy problems. In this way, patients, rather than health care providers, are responsible for deciding whether or not to provide information to others. However, this method is only suitable for patients with sufficient cognitive abilities and may not be useful in crisis situations. Moreover, as was argued before, patients' desire to protect their privacy by concealing their records or parts of it, may cause health care providers not to have access to relevant patient information and may thereby negatively affect the quality of care.

### Reliability and quality of patient information

With respect to the reliability and quality of patient information, inadequate or incomplete recording of patient data was perceived as an important threat to successful electronic information exchange. This may cause essential information to be missed or misunderstood, and may thereby negatively affect the quality of care. Although recent guidelines [e.g. [12]] focus on the importance of good quality recording, the application of these guidelines in daily practice is limited [22]. This may partly be caused by the limited comprehensibility and usefulness of these guidelines for daily practice. Developing user-friendly summaries of guidelines and training health care providers in applying them is important to improve recording behaviours and thereby enhance the uniformity and quality of patient records [cf. [22]].

Such improvements can also be achieved by adapting electronic information systems to facilitate adequate recording (e.g. including effective and efficient search functions), and by regularly evaluating health care providers' records and providing feedback. Several countries have developed standardised tools (e.g. the EMR scan in the Netherlands and PRIMIS+ in England) to evaluate the quality of recording by general practitioners and to provide feedback [23-25]. Until now however, the Dutch EMR scan has merely been implemented in a few regions. Implementation on a larger scale may improve the quality of recording.

Technical problems, such as the limited interoperability between information systems and the limited speed of electronic information exchange were also perceived as threats to the reliability and quality of electronically exchanged patient information [cf. [1,2]]. As can be expected considering the nature of the health care setting, problems regarding the speed of electronic information exchange were mainly reported in acute care. Because of these technical problems, essential information may be unavailable during consultations. This can adversely affect the quality and efficiency of care, but also complicates liability issues. By improving the interoperability between electronic information systems, patient information may be imported directly into the records of receiving health care providers, which would increase the efficiency of information exchange.

Health care providers in ambulatory mental health care perceived the large amount of patient information being available by electronic information exchange as a problem, since this could cause them to miss essential information. To prevent the overload of information being exchanged in this health care setting, a summary care record may be introduced, comparable to the electronic locum record used in the information exchange between GPs and out-of-hours services, which includes only the most essential patient information (e.g. current medication use and information on allergies and intolerances). This requires consensus within the mental health care setting about the patient information to be included in the summary care record.

Finally, respondents perceived the unfamiliarity of other health care providers as a problem of electronic information exchange in case of an n-EPR. They had more trust in information received from colleagues they already knew. Several respondents indicated that they were familiar with health care providers in the region and therefore expressed a preference for regional systems of electronic information exchange over an n-EPR. Whether health care providers actually know every colleague in the region can be questioned, however. Organising meetings between health care providers who regularly exchange information may be useful to build trust.

### Methodological reflections

This study was conducted in three health care settings. To obtain a broad picture of health care providers' perceptions of electronic information exchange, we aimed to include two health care organisations in each setting that differed in the extent to which electronic information exchange was being used. This aim was not met for ambulatory mental health care.

Because our study included a limited number of health care providers and settings, caution should be exercised in generalising the findings. Respondents with explicit opinions about electronic information exchange and the n-EPR may have been more likely to participate in this study, thereby possibly biasing the results, either positively or negatively. To minimise this effect, interview questions addressed perceived problems as well as benefits of electronic information exchange.

Because of its cross-sectional design, the study did not take into account the longitudinal character of the implementation process. Previous research has shown that health care providers' resistance towards the implementation of information technology changes over time [19]. Early in the implementation process, the object of resistance is the technology itself and its features, whereas in later stages, resistance becomes more politicised and is focused at the significance of the new technology or its advocates. Future research should investigate which strategies can best be used to promote the adoption of the n-EPR in various stages of the implementation process.

## Conclusions

Implementation of electronic information exchange in health care is a complex process, which requires changes at various levels [16,26,27]. The n-EPR can only be implemented successfully if top-down macro level changes (e.g. in legal regulations and technical infrastructure) are combined with changes at the level of its users. Because health care providers' perceptions about electronic information exchange are likely to affect their willingness to adopt the n-EPR, addressing their needs and preferences is crucial.

## Competing interests

The authors declare that they have no competing interests.

## Authors' contributions

MZ and FW carried out the data collection. MZ performed the analyses and drafted the manuscript. RV and RF designed the study and formulated the research questions. All authors critically reviewed the manuscript. All authors read and approved the final manuscript.

## Pre-publication history

The pre-publication history for this paper can be accessed here:

http://www.biomedcentral.com/1472-6963/11/256/prepub

## Supplementary Material

Additional file 1 **The Dutch n-EPR**. Description of the characteristics and implementation process of the Dutch n-EPR. Click here for file

Additional file 2 **Topic list used in the interviews**. List of topics used in the interviews. Click here for file
