# Supplementary material for: Benefits and problems of electronic information exchange as perceived by health care professionals: an interview study
Source: BMC Health Serv Res. 2011 Oct 7;11:256. doi: 10.1186/1472-6963-11-256 (PMC3200179; doi:10.1186/1472-6963-11-256)
Supplement: Additional file 1 — The Dutch n-EPR. Description of the characteristics and implementation process of the Dutch n-EPR. [file 1472-6963-11-256-S1.DOC]

## Additional file 1 - The Dutch n-EPR

| The Dutch n-EPR can best be regarded as a referral system, through which a selection of medical data (summary care record) stored in local electronic patient records are in principle accessible for specific groups of health care providers. Patient data will not be stored in a central database, but will be exchanged through the National Switch Point (NSP). The NSP holds an index record for each Dutch inhabitant, indicating where his/her electronic patient record is stored. Once the n-EPR has been fully implemented, patient data will be available to authorised medical professionals at any moment from every health care practice, by making use of the patient’s index record.  Before being allowed to connect to the national infrastructure, health care organisations have to fulfil a set of organisational, technical and security requirements.  To provide safe and reliable electronic information exchange, patients and healthcare professionals need to identify themselves electronically. The Citizen Service Number, which is unique for each Dutch citizen, is used for linking electronic patient records to individual patients. Health care providers can get access to the n-EPR by means of a personal ID chip card (the so-called UZI pass, which is a Dutch abbreviation for ‘Unieke Zorgverlener Identificatie pas’: ‘unique health care provider identification pass’) and password. The access to patient records is restricted to health care providers with a legitimate relationship to the patient (role based access). All accesses are logged.  Patients have the right to inspect their own record and to conceal, change or delete their record or parts of it. They also have the right to inspect the logging data of their record to see which health care providers have accessed their record.  The implementation of the Dutch n-EPR is meant to follow a stepwise approach. First, electronic information exchange between GPs and out-of-hours services and the exchange of information about medication between GPs, medical specialists and pharmacists will be implemented, possibly followed by implementation in other segments of health care (e.g. in emergency care or of in the exchange of laboratory data). In September 2010, percentages of health care organisations connected to the NSP varied from 14% for general practices to 54% for out-of-hours services [1].  The Dutch government has used several strategies which aimed to contribute to the implementation of the n-EPR. Firstly, the legal framework for the Dutch n-EPR obliged health care providers to connect to the national infrastructure. Financial incentives were provided to health care providers to stimulate this development. By early implementation of the n-EPR in some regions, the government aimed to provide some positive examples and thereby persuade others to follow. Information about the necessity of the n-EPR was provided to health care providers and patients by means of public media campaigns, letters and a website. |
| --- |

# References

1. Schippers EI: *Voortgangsrapportage landelijke infrastructuur voor gegevensuitwisseling in de zorg, derde kwartaal 2010* [Progress report national infrastructure for information exchange in health care, third quarter of 2010]. The Hague: Ministry of Health, Welfare and Sport; 2010
