# Supplementary material for: Benefits and problems of electronic information exchange as perceived by health care professionals: an interview study
Source: BMC Health Serv Res. 2011 Oct 7;11:256. doi: 10.1186/1472-6963-11-256 (PMC3200179; doi:10.1186/1472-6963-11-256)
Supplement: Additional file 2 — Topic list used in the interviews. List of topics used in the interviews. [file 1472-6963-11-256-S2.DOC]

## Additional file 2 - Topic list used in the interviews

| **Scenario describing a patient’s contact with the health care organisation, used in diabetes care** |
| --- |
| Mr. B. (aged 70) consults the GP with complaints of fatigue, thirst and shortness of breath. After a blood test, the GP diagnoses diabetes mellitus type 2. He prescribes Mr. B medication, which can be obtained from the pharmacy. The GP also refers Mr. B. to a dietician. After three months, Mr. B. visits the general practice for a regular check-up. A GP nurse executes the physical examination and discusses the results with the GP. For the yearly check-up, the GP refers Mr. B. to a podotherapist and an oculist. The GP receives the results of the examinations of these health care providers. Based on these results, Mr. B. is being referred to a specialist in internal medicine for additional examination and treatment. |
| **Interview questions** |
| The scenario includes various occasions in which information is being exchanged between health care providers. Please answer *for each of these occasions* how this is handled in your health care organisation:   - In what way is the information being exchanged? (on paper, by telephone, fax etc.) Are there exceptions to the processes described above? - Are there any problems in this process? If so, how could these problems be prevented? - Do you trust the quality of the information that is being exchanged? Why or why not? - Do you trust the safety of information exchange? Why or why not? |
| Legal aspects of information exchange   - Are there any protocols or guidelines to guide electronic information exchange in your health care organisation? - Are health care providers familiar with these protocols and guidelines? If not, why not? - Are the protocols and guidelines being used by health care providers? If not, why not? |
| N-EPR   - Do you feel the need for a national system? Why or why not? - Do you trust the quality of the information that is being exchanged through the n-EPR? Why or why not? - Do you trust the safety of information exchange through the n-EPR? Why or why not? |
| Factors potentially influencing trust in electronic information exchange   - Which factors affect your trust in electronic information exchange at a regional level? (e.g. legal, organisational, practical or technical factors). For each of the factors mentioned to negatively affect trust: what could be done to improve your trust? - Which factors affect your trust in electronic information exchange at a national level? (e.g. legal, organisational, practical or technical factors). For each of the factors mentioned to negatively affect trust: what could be done to improve your trust? |
| - What do you perceive to be the most important benefits of electronic information exchange? - What do you perceive to be the most important risks of electronic information exchange? |
